# Genetic Mutations and Small Bowel Ulcerating Disease: Role in Diagnosis?

**DOI:** 10.1007/s11894-025-00978-4

**Published:** 2025-05-21

**Authors:** Rangesh Modi, Tanner Storozuk, Namrata Setia

**Affiliations:** 1 Department of Internal Medicine, Section of Gastroenterology, Hepatology, and Nutrition, University of Chicago, Chicago, IL 60637 USA; 2 Department of Pathology, University of Chicago, 5841 S Maryland Ave, E607, Chicago, IL 60637 USA

**Keywords:** Small Bowel Ulcers, Crohn’s disease, NSAID enteropathy, Genetics, Polymorphisms, CMUSE, CNSU

## Abstract

**Purpose of Review:**

This review examines the role of genetic variations in the pathogenesis of small bowel (SB) ulcers associated with Crohn's disease (CD), NSAID enteropathy, and Cryptogenic Multifocal Ulcerous Stenosing Enteritis (CMUSE)/Chronic Non-Specific Ulcers of the Small Intestine (CNSU), aiming to address current diagnostic challenges.

**Recent Findings:**

Advances in molecular genetics have revealed significant genetic contributors to SB ulceration. In CD, the *NOD2* gene on chromosome 16 and several additional risk variants identified through genome-wide association studies (GWAS)—with key insights from the International Inflammatory Bowel Disease Genetics Consortium—have enhanced our understanding of the pathobiology of the disease. In NSAID enteropathy, polymorphisms in CYP enzymes have been associated with altered drug metabolism and gastrointestinal complications. However, the genetic mechanisms underlying deep ulcers in NSAID enteropathy, as well as CMUSE/CNSU, remain poorly understood.

**Summary:**

Genetic insights are crucial for understanding SB ulcerative diseases. Future research should focus on identifying specific genetic determinants to improve diagnostic accuracy and therapeutic strategies.

## Introduction

Small bowel ulcerating diseases, including Crohn's disease (CD), NSAID enteropathy, and rare disorders like Cryptogenic Multifocal Ulcerous Stenosing Enteritis (CMUSE) & Chronic Non-Specific Ulcers of the small intestine (CNSU), pose significant challenges to clinicians due to their complex and multifactorial nature. These conditions often present with overlapping clinical and histologic features, complicating both diagnosis and management. Recent advances in molecular genetics have highlighted the role of genetic variations in the development of small bowel ulceration, offering fresh perspectives on the underlying mechanisms and potential diagnostic approaches. This paper sequentially reviews the evolving role and available literature on the genetic variations in CD, NSAID enteropathy, and CMUSE/CNUSE.

### Small Bowel Crohn’s Disease

Although small bowel (SB) is involved in 30–70% patients with CD, isolated SB CD occurs in only 30% of individuals [1]. Patients with isolated SB CD have higher incidence of stricturing and severity of inflammation compared to those with both small and large bowel involvement [2]. CT or MR enterography (CTE or MRE) of SB CD usually demonstrates segmental stratified mural or asymmetric mural enhancement, bowel wall thickening and edema [3]. Complications such as long segment stricture, fistula, and abscess can differentiate SB CD from other etiologies [3]. Mesenteric lymphadenopathy and hypervascularity are other findings typical for CD [3]. Apart from the findings caused by complications of CD, most features on imaging are nonspecific and can occur in other etiologies of SB ulcers/strictures. Intestinal Ultrasound (IUS) in SB CD shows increased wall thickness, vascularization, and complications such as stricture with good sensitivity and specificity in established rather than suspected CD [4]. However, similar findings were reported in a case of NSAID associated SB ulcer/stricture [5]. No large studies exist to date to determine the utility and reliability of IUS in reliably differentiating SB CD from other etiologies.

**Fig. 1:**
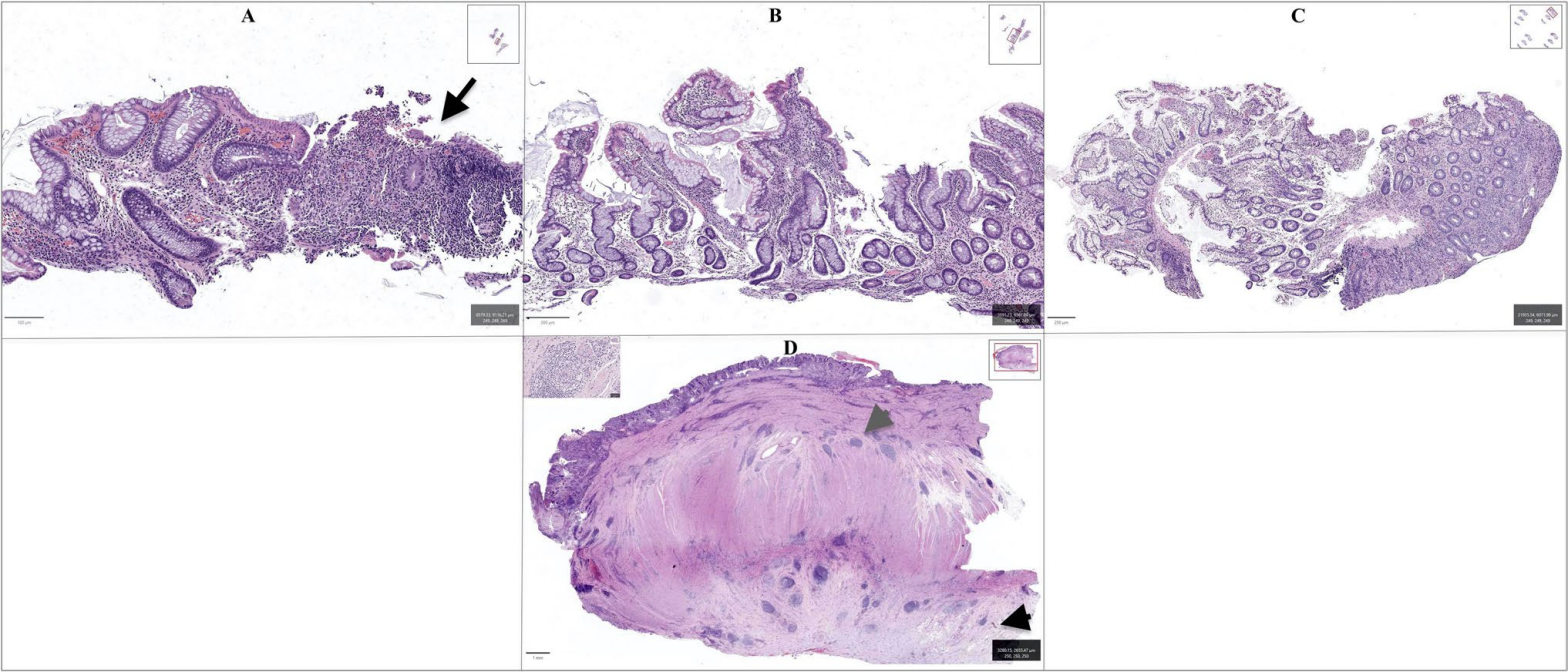
Biopsies from Crohn’s disease enteritis (image **A**), NSAID enteropathy (image **B**), and CMUSE/CNSU (image **C**) show a range of changes, including architectural distortion observed across all biopsies. However, aphthous erosions (black arrow, image **A**) and a more pronounced degree of lamina propria inflammation are characteristic of Crohn’s disease. Histology of CMUSE and CNSU is nonspecific with varying intensity of inflammation as seen in image **C**. The pathognomonic diagnostic features of active Crohn’s disease are most evident in resection specimens (image **D**), which demonstrate transmural inflammation (grey arrowhead), granulomas (inset, image **D**), and serosal adhesions (black arrowhead)

Capsule endoscopy (CE) is being increasingly utilized for SB CD. Based on findings of ulcer, extent of inflammation, villous appearance, etc. few scoring systems like Lewis score, and Crohn’s activity index are devised [6]. However, diagnostic yield for CE in active SB CD is similar to MRE [6]. Slower transit of capsule can occur especially in active CD due to diminished gut motility rather than strictures [6]. Device assisted enteroscopy (DAE) allows direct visualization of SB, tissue sampling, and endoscopic therapy [6]. A study involving 44 patients who underwent DAE after CE for SB CD showed that in an additional 9% of patients CD was excluded and 14% of patients the disease was confirmed [7]. Another retrospective study looking at diagnostic yield for double-balloon enteroscopy (DBE) showed that despite positive macroscopic findings from DBE, 58% of the patients had normal or nonspecific histology, and 45% of patients were treated as having CD on the basis of a combination of histology, endoscopic appearance, clinical symptoms, and laboratory tests [8]. Histologically, CD shows inflammation that is transmural, and granulomatous with complications including strictures, fistulas, abscess formation, and dysplasia/neoplasia. On biopsy (with sampling limited to the surface mucosa), chronic enteritis with focal activity and/or ulceration can be seen. Chronic changes, which are not specific to Crohn’s disease (CD), including crypt architectural distortion and pyloric gland metaplasia, are often present. The degree of lamina propria inflammation is greater than what is typically observed in NSAID enteropathy. While not consistently identified, the presence of non-caseating granulomas, when present, can be a useful histologic feature in supporting a diagnosis of CD over other etiologies (Fig. 1). Although diagnostic techniques have improved significantly over the past few decades, small bowel endoscopy and deep tissue histology are still insufficient for reliably diagnosing SB CD in all cases. This can result in the inappropriate use of biologics and multiple small bowel surgeries, often leading to short bowel syndrome (SBS), highlighting the need for additional diagnostic modalities that offer greater specificity.

### Genetic of Crohn’s Disease

The pathophysiology of IBD remains poorly understood but involves a complex interplay between genetics, epigenetics, environmental factors, intestinal flora, and aberrant host immune responses [9]. Despite these complexities, family studies have highlighted a genetic component in CD, with a greater degree of familial aggregation observed among affected individuals. Genetic linkage studies, which examine multiple affected families to pinpoint broad chromosomal regions associated with disease, and then refine these to specific genetic loci, have identified the pericentromeric region of chromosome 16 as a critical locus related to CD, mainly due to the *NOD2* gene [10]. This discovery has been validated by independent research groups and has proven instrumental in advancing our understanding of the genetic underpinnings of CD. Furthermore, genome-wide association studies (GWAS) have identified additional genetic variants linked to CD, offering a more complete picture of the genetic factors contributing to the disease. These studies collectively underscore the significant genetic influence in CD, especially when compared to UC. This review will summarize genetic variants identified as significant in the recent meta-analysis conducted by the International Inflammatory Bowel Disease Genetics Consortium (IIBDGC, https://www.ibdgenetics.org/). The IIBDGC is a global network of hundreds of researchers from over 20 countries across four continents, with the aim of uncovering genetic risk factors and their interactions in IBD, including CD, UC, and related clinical phenotypes.

The group’s first publication in 2001 provided compelling evidence for genetic linkage among CD families in the pericentromeric region of chromosome 16, also known as the *IBD1* locus [11]. Subsequent studies by independent groups have confirmed that the observed linkage in this region is attributable to the *NOD2* gene [12, 13]. Specifically, three coding region variants in the *NOD2* gene—p.L1007fsinsC/c.3020insC, p.R702W/c.2104C>T, and p.G908R/c.2722G>C—are associated with an increased susceptibility to CD, but not UC. The L1007fsinsC/3020insC variant, in particular, is linked to impaired LPS-induced NFκB activation, in contrast to the activity observed in the wild-type *NOD2*, suggesting a direct role in the pathophysiology of CD. *NOD2*, a cytosolic protein predominantly expressed in monocytes, is involved in bacterial sensing, and a deficiency in this function may lead to an exaggerated inflammatory response by the immune system. This dysregulated immune response, potentially affecting cytokine production such as interleukin- 10, could underlie the increased susceptibility to CD in individuals carrying these *NOD2* variants.

Subsequent GWAS studies identified additional SNPs strongly associated with CD beyond *NOD2* [14]. One such SNP located in an intron of *TNFSF15*, which encodes tumor necrosis factor superfamily member 15, was found to be associated with CD in Japanese population. A North American study, which included over 1,000 CD cases and controls, reported a strong association between multiple SNPs in the *interleukin 23 receptor* gene (IL23R) and CD. Notably, the rare, non-synonymous SNP p.R381Q (rs11209026) showed the strongest association and a protective effect. Another non-synonymous SNP, p.T300A (rs2241880), located in the *autophagy-related 16-like 1* gene (*ATG16L1*), was associated with CD in German and British populations. Additionally, a scan of Belgian CD cases identified an association with a 1.2-Mb region on chromosome 5p13.1, a gene desert implicated in the regulation of the *prostaglandin E receptor 4 gene* (*PTGER4*), which encodes the prostaglandin receptor EP4.

Further studies have fine-mapped both previously reported and new loci implicated in CD genetics. Huang et al*.* genotyped 67,852 individuals of European ancestry, including 33,595 with inflammatory bowel disease (IBD) (18,967 with CD and 14,628 with UC) and 34,257 healthy controls, using the Illumina ImmunoChip [15]. To perform fine mapping, the authors conducted stringent quality control to eliminate genotyping errors and batch effects. They found that 68 of the 94 regions contained a single association, while 26 regions harbored two or more independent signals, resulting in a total of 139 independent associations across the 94 regions. Only *IL23R* and *NOD2* (both of which had previously been shown to contain multiple associated protein-coding variants) contained more than three independent signals. Of the 139 signals, 79 were more strongly associated with CD, 23 with UC, and 37 were equally associated with both subtypes. The authors estimated that the reported associations explained approximately 25% of the risk for CD. The single-variant credible sets included five previously reported coding variants: three in *NOD2* (fs1007insC, p.R702W, p.G908R), a rare protective allele in *IL23R* (p.V362I), and a splice variant in *CARD9* (c.IVS11+1G>C). The remaining single-variant credible sets comprised three missense variants (p.I170V in *SMAD3*, p.I923V in *IFIH1*, and p.N289S in *NOD2*), four intronic variants (in *IL2RA*, *LRRK2*, *NOD2*, and *RTEL1/TNFRSF6B*), and six intergenic variants (located 3.7 kb downstream of *GPR35*, 3.9 kb upstream of *PRDM1*, within an EP300 binding site 39.9 kb upstream of *IKZF1*, 500 bp before the transcription start site of *JAK2*, 9.4 kb upstream of *NKX2 - 3*, and 3.5 kb downstream from *HNF4A*). The authors also noted that all common coding variants previously reported to affect IBD risk were included in their 95% credible set. These variants included those in *IL23R* (p.R381Q, p.V362I, p.G149R), *CARD9* (c.IVS11+1G>C and p.S12N), *NOD2* (p.S431L, p.R702W, p.V793M, p.N852S, p.G908R, fs1007insC), *ATG16L1* (p.T300A), *PTPN22* (p.R620W), and *FUT2* (p.W154X). This enrichment of coding variants suggests that these are likely to confer  stronger effect sizes.

Polygenic risk scores (PRS) proposed by Cho et al*.* have been published to predict inflammatory bowel disease (IBD) risk using exome-sequencing and SNP array data from 29,358 individuals in the multi-ethnic, randomly ascertained health system-based BioMe biobank [16]. The authors employed regression models to predict IBD status, similar to the approach used in diabetes genetic risk studies, combining risk scores with gender, age, smoking status, and minor allele counts for top IBD-associated SNPs. These top IBD-associated SNPs included *NOD2* p.R702W (rs2066844), *NOD2* p.G908R (rs2066845), *NOD2* p.L1007fs (rs2066847), *IL23R* p.R381Q (rs11209026, protective), *LRRK2* p.R1398H (rs7133914, protective), and *LRRK2* p.N2081D (rs33995883). The results of this study are more directly applicable to CD rather than IBD in general, as the cohort primarily consisted of CD patients. Specifically, 311 out of 339 total IBD patients in the BioMe Biobank were diagnosed with CD. Moreover, datasets focusing on Ashkenazi Jewish and African American populations were also centered around CD cases, further reinforcing that the findings are particularly relevant to CD.

Compared to prior meta-analyses, the most recent study by Sazonovs et al*.* performed a large-scale exome sequencing analysis using CD case and control collections from over 35 centers in the IIBDGC [17]. The primary goal was to complement previous GWAS and better define actionable biological targets by identifying the low-frequency and rare coding variants in the genetic architecture of CD. The analysis involved exome sequencing of 30,036 Crohn’s disease cases and 80,988 controls from different cohorts. The authors identified 45 variants that exceeded the study-wide significance threshold. This included 8 variants that reached exome-wide significance in genes such as *NOD2*, *IL23R, TYK2*, *PTPN22*, and *CARD9*, as well as 6 variants in *GPR65*, *MST1*, *NOD2*, and *SMAD3* with genetic effects consistent with those previously reported. Five novel coding variants in genes not previously implicated in IBD were identified, along with 6 independently associated novel exonic variants in genes previously known to harbor mutations underpinning CD or IBD risk (2 of which were in *NOD2*). The study also recaptured 14 significant variants in known IBD causal candidates, including variants in *CARD9*, *IL23R*, and *NOD2*. The remaining 20 variants either tagged known causal variants through linkage disequilibrium or had very small posterior inclusion probabilities (PIP) from fine-mapping, making them highly unlikely to be causal for CD. The list of these variants is provided in Table 1. This study identified several newly implicated genes in Crohn's disease (CD) risk, including *DOK2*, involved in macrophage and T cell signaling and inflammation; *TAGAP*, linked to T-cell activation and Th17 development; *PTAFR*, regulating colitis-induced pulmonary inflammation; *PDLIM5*, interacting with *STAT3* and expressed in intestinal myofibroblasts; *SDF2L1*, supporting gut barrier function and mucus integrity; *CCR7*, regulating immune cell homing and inflammation; *IL10RA*, associated with early-onset IBD; *RELA*, influencing immune responses via NF-κB; *ATG4C*, involved in autophagy; and *HGFAC*, regulating neutrophil recruitment and epithelial regeneration in intestinal inflammation.

**Table 1:** List of 45 exome significant variants reported by Sazonovs *et al* [17]

SNP^†^ ID	Chr	Position	A0	A1	Variant	gnomad_non-Finnish European_AlleleFrequency	Status	Consequence	Gene	p./c. nomenclature
rs141992399	9	136,365,140	C	G	9–136,365,140-C-G	0.005366	Known causal candidate	splice donor variant	*CARD9*	c.1434+1G>C
rs2228015	17	40,558,934	T	C	17–40,558,934-T-C	0.039339	New Locus	start loss	*CCR7*	M7V
rs34215892	8	21,909,729	G	A	8–21,909,729-G-A	0.031608	New Locus	missense variant	*DOK2*	P274L
rs16844401	4	3,447,925	G	A	4–3,447,925-G-A	0.070423	New variant in known locus	missense variant	*HGFAC*	R516H
rs56143179	11	117,998,788	C	T	11–117,998,788-C-T	0.0014248	New Locus	missense variant	*IL10RA*	P295L
rs41313262	1	67,240,217	G	A	1–67,240,217-G-A	0.016717	Known causal candidate	missense variant	*IL23R*	V362I
rs11209026	1	67,240,275	G	A	1–67,240,275-G-A	0.058635	Known causal candidate	missense variant	*IL23R*	R381Q
rs104895431	16	50,711,203	C	T	16–50,711,203-C-T	0.0016687	Known causal candidate	missense variant	*NOD2*	S431L
rs104895438	16	50,711,745	G	A	16–50,711,745-G-A	0.00024624	Known causal candidate	missense variant	*NOD2*	A612T
rs2066844	16	50,712,015	C	T	16–50,712,015-C-T	0.043291	Known causal candidate	missense variant	*NOD2*	R702W
rs5743277	16	50,712,018	C	T	16–50,712,018-C-T	0.0054909	Known causal candidate	missense variant	*NOD2*	R703C
rs61747625	16	50,712,175	C	T	16–50,712,175-C-T	0.0043503	Known causal candidate	missense variant	*NOD2*	A755V
rs104895467	16	50,716,899	A	G	16–50,716,899-A-G	0.00083502	Known causal candidate	missense variant	*NOD2*	N852S
rs2066845	16	50,722,629	G	C	16–50,722,629-G-C	0.01427	Known causal candidate	missense variant	*NOD2*	G908R
rs2066847	16	50,729,867	G	GC	16–50,729,867-G-GC	0.022959	Known causal candidate	frameshift variant	*NOD2*	fs1007insC
rs104895443	16	50,712,243	G	A	16–50,712,243-G-A	0.0004844	New variant in known locus	missense variant	*NOD2*	E778K
rs748508924	16	50,716,671	G	GT	16–50,716,671-G-GT	0.00021125	New variant in known locus	splice donor variant	*NOD2*	c.2465+2dup
rs182339387	4	94,573,345	C	T	4–94,573,345-C-T	0.0034261	New Locus	splice region variant	*PDLIM5*	c.249-6C>T
rs138629813	1	28,150,681	T	C	1–28,150,681-T-C	0.0036568	New Locus	missense variant	*PTAFR*	N114S
rs61759893	11	65,658,293	C	T	11–65,658,293-C-T	0.0057034	New variant in known locus	missense variant	*RELA*	D291 N
rs73166641	22	21,643,991	G	A	22–21,643,991-G-A	0.015208	New variant in known locus	missense variant	*SDF2L1*	R161H
rs13107325	4	102,267,552	C	T	4–102,267,552-C-T	0.06954	Known causal candidate	missense variant	*SLC39A8*	A391T
rs41267765	6	159,041,392	C	T	6–159,041,392-C-T	0.023316	New variant in known locus	missense variant	*TAGAP*	E147K
rs34536443	19	10,352,442	G	C	19–10,352,442-G-C	0.041674	Known causal candidate	missense variant	*TYK2*	P1104A
rs35018800	19	10,354,167	G	A	19–10,354,167-G-A	0.0080175	Known causal candidate	missense variant	*TYK2*	A928V
rs10065172	5	150,848,436	C	T	5–150,848,436-C-T	0.090036	Unlikely causal	synonymous variant	*IRGM*	L105L
rs142408856	9	136,464,447	C	T	9–136,464,447-C-T	0.025854	Unlikely causal	synonymous variant	*SEC16A*	P1473P
rs7565275	2	233,320,674	A	G	2–233,320,674-A-G	0.050955	Unlikely causal	missense variant	*SAG*	I76V
rs104895423	16	50,710,654	T	G	16–50,710,654-T-G	0.00054538	Unlikely causal	missense variant	*NOD2*	L248R
rs34569521	9	136,381,955	C	T	9–136,381,955-C-T	0.083372	Unlikely causal	missense variant	*SNAPC4*	R729Q
rs36075906	2	233,527,423	C	T	2–233,527,423-C-T	0.046133	Unlikely causal	missense variant	*USP40*	R581Q
rs104895421	16	50,699,481	T	A	16–50,699,481-T-A	0.001373	Unlikely causal	splice region variant	*NOD2*	c.74-7T>A
rs74017766	16	50,669,105	C	T	16–50,669,105-C-T	0.011268	Unlikely causal	missense variant	*SNX20*	R109H
rs2228058	5	40,681,152	C	T	5–40,681,152-C-T	0.073406	Unlikely causal	synonymous variant	*PTGER4*	T53T
rs13011156	2	233,263,166	A	G	2–233,263,166-A-G	0.061779	Unlikely causal	synonymous variant	*ATG16L1*	L82L
rs113009360	1	67,206,980	T	C	1–67,206,980-T-C	0.0070673	Unlikely causal	synonymous variant	*IL23R*	Y241Y
rs11175964	12	40,309,185	G	A	12–40,309,185-G-A	0.072084	Unlikely causal	synonymous variant	*LRRK2*	K1423K
rs7133914	12	40,309,109	G	A	12–40,309,109-G-A	0.072712	Unlikely causal	missense variant	*LRRK2*	R1398H
rs7308720	12	40,263,898	C	G	12–40,263,898-C-G	0.07203	Unlikely causal	missense variant	*LRRK2*	N551K
rs2066852	16	50,793,607	C	T	16–50,793,607-C-T	0.074559	Unlikely causal	synonymous variant	*CYLD*	D804D
rs104895447	16	50,716,931	A	G	16–50,716,931-A-G	0.0011514	Unlikely causal	missense variant	*NOD2*	M863V
rs33995883	12	40,346,884	A	G	12–40,346,884-A-G	0.019446	Unlikely causal	missense variant	*LRRK2*	N2081D
rs79124041	16	50,668,046	G	A	16–50,668,046-G-A	0.032483	Unlikely causal	splice region variant	*SNX20*	c.*995C>T
rs5743291	16	50,723,365	G	A	16–50,723,365-G-A	0.088485	Unlikely causal	missense variant	*NOD2*	V955I
rs201586544	16	50,699,554	C	T	16–50,699,554-C-T	0.00032589	Unlikely causal	missense variant	*NOD2*	S47L

^†^Single Nucleotide Polymorphism

While the referenced studies primarily address genetic variants implicated in CD, rather than IBD in general, the details regarding whether the disease initially involve the small bowel or other regions of the GI tract, or it over time, remain unclear in the majority of the cited literature. Furthermore, several deep intronic variants have been associated with a predisposition to CD, review of which fall outside the scope of this manuscript. It is recommended that readers refer to additional publications from the IIBDGC (https://www.ibdgenetics.org/) for further information.

## NSAID Enteropathy

Non-steroidal anti-inflammatory drugs (NSAIDs) are one of the commonly used prescription as well as over-the-counter medicines worldwide. According to a study, 30 million people worldwide take NSAIDs every day, and NSAIDs have a prescription cost of 111 million per year in the United States, valued at approximately 480 million dollars [18]. The primary mechanism of action is the inhibition of the cyclooxygenase (COX) enzyme that produces various prostaglandins (PG) from the cleavage of precursor arachidonic acid [19]. However, the same reduction in PGs triggers their known cardiovascular, renal, and gastrointestinal (GI) toxicities [19].

The majority of GI toxicity is limited to upper gastrointestinal (gastric and duodenal) ulceration/bleeding. However, the advent of capsule endoscopy and balloon-assisted enteroscopy has helped uncover the small bowel toxicities associated with NSAID use. Intestinal injuries from NSAIDs, such as inflammation, mucosal erosion, increased mucosal permeability, ulceration, perforation as well as death, have been described in the literature [20]. Concentric diaphragmatic strictures occur as a secondary complication due to scarring and fibrosis with chronic NSAID use. Hence, the term Diaphragm disease is used to denote NSAID-associated small bowel strictures [21]. In a study examining 120 patients on long-term NSAID use, capsule endoscopy estimated the prevalence of diaphragm-like strictures to be 2% [22]. However, according to another study for patients taking non-selective NSAIDs for more than 3 months, the prevalence of small bowel injury was 71% [23]. The exact prevalence of small bowel ulcers remains unknown to date.

NSAID associated small bowel ulcers and strictures are often difficult to distinguish on endoscopy, radiology, and histology. The current diagnostic criteria for NSAID enteropathy rely on the history of NSAID use and the exclusion of other etiologies [24]. Histologic examination of the biopsy specimens typically shows an active and/or chronic enteritic pattern of injury with/without ulceration. Crypt architectural distortion and pyloric metaplasia may be seen, but are more commonly seen in CD. The degree of lamina propria lymphocytic inflammation is often less than what is expected in CD. However, these changes are not entirely specific posing a diagnostic challenge [Fig. 1]. NSAID-associated small bowel injury can last up to 18 months post discontinuation, but studies are limited for this data [24]. Management remains surgical resection, endoscopic dilation/therapy, and discontinuing NSAIDs. However, no specific medications have been tested in larger randomized trials to treat NSAID enteropathy.

### Genetics of NSAID Injury

Cytochrome P- 450 (CYP) is a heme protein involved in the metabolism of drugs and other xenobiotics in the human body [25]. The human genome has 57 *CYP* genes divided into further subfamilies [26]. The major metabolizing isoforms are CYP2C9, CYP1A2, CYP2C19, CYP2D6 and CYP3A4 [26]. Among these, *CYP2C9* is known to have many genetic polymorphisms and is extensively involved in NSAID metabolism [26]. To date, more than 80 *CYP2C9* allelic variants have been identified [26]. The most commonly reported variants identified linked to reduced NSAID metabolism, and gastrointestinal (GI) side effects are CYP2C9*2 denoted by rs1799853 (c.430 C>T, p.R144 C) associated with decreased function of CYP2C9, and CYP2C9*3 denoted by rs1057910 (c.1075A>C, p.I359L) associated with no function of CYP2C9 enzyme.

Within the field of Gastroenterology and Hepatology, genetic studies have been performed on NSAID-associated liver toxicity, effect on adenoma recurrence, risk of peptic ulcer, and upper GI bleeding. No genetic studies have been performed to date on NSAID-induced deep small bowel ulcers and strictures. For the scope of this review, we will not include studies on genetics affecting adenoma prevention risk and hepatotoxicity by NSAIDs. Table 2 summarizes the below-mentioned studies for NSAID-associated GI tract ulceration/bleeding [27].

**Table 2:** Summary of studies on NSAID associated ulcers/bleeding

Study	Design	Patient characteristics	NSAID types	Key outcomes
Pilotto et al. [27]	Retrospective study	26 patients (*H. pylori* excluded) with NSAID-related upper GI bleeding confirmed via endoscopy	Diclofenac (11), celecoxib (5), piroxicam (5), naproxen (2), ibuprofen (3)	CYP2C9*1/3 and CYP2C9*1/2 genotypes associated with risk
Martinez et al. [28]	Case–control study comparing NSAID-related upper GI bleeders and NSAID users without adverse effects	Patients characterized according to taking NSAIDs extensively metabolized by *CYP2C9* vs. partially metabolized	Celecoxib (3), diclofenac (8), ibuprofen (9), lornoxicam (1), piroxicam (7), aceclofenac (2), indomethacin (1), naproxen (2)	Variant allele carriers had higher bleeding risk (OR 2.60; 95% CI 1.10–6.19). CYP2C9*2 associated with trend of increased bleeding
Figueiras et al. [29]	Multi-center case–control study involving European patients	Cases (577): Upper GI bleeding confirmed via endoscopy Controls (1343): Preoperative patients undergoing minor non-NSAID-related surgery*. pylori* infection excluded via serology	Celecoxib, diclofenac, ibuprofen, naproxen, aceclofenac, indomethacin, lornoxicam, piroxicam	CYP2C9*3 was strongly associated with GI bleeding risk at higher NSAID doses (> 0.5 DDD). No association with CYP2C9*2
Kraus et al. [30]	Subset analysis of PreSAP trial participants, focusing on genetic polymorphisms and GI toxicity from celecoxib	117 Israeli patients who had undergone colonoscopy for adenoma	Celecoxib; placebo control group also included	Genetic polymorphisms in *EGFR*, *ALOX15*, and *PTGEP4* genes linked to GI toxicity
Shiotani et al. [31]	Case–control study of patients on aspirin (100 mg)	68 patients with peptic ulcer (20 with bleeding ulcers) and 357 controls	Low-dose aspirin (100 mg) for cardio protection	*AGT*− 20CC genotype significantly associated with bleeding ulcers *AT1R*− 521CC genotype associated with peptic ulcers in subgroup without ACE inhibitor/ARB co-treatment
Shiotani et al. [32]	Case–control study on aspirin-associated small bowel bleeding	37 cases (small bowel bleeding) and 400 controls	Low-dose aspirin (100 mg)	*CYP2D6 − 2178GG* (rs28360521, *CYP2D6* c.− 2178G>A) homozygous genotype independently associated with increased risk of small bowel bleeding
Tatarunas et al. [33]	Prospective study of patients hospitalized for acute coronary syndrome treated with antiplatelet therapy	6 months of antiplatelet therapy (clopidogrel, ticagrelor, and aspirin). Assessed non-procedural bleeding (nosebleeds, GI, or GU). Endoscopic confirmation of GI bleeding not provided	Aspirin (300 mg loading dose and 81 mg daily thereafter)	*CYP4 F2 T* allele (rs3093135, *CYP4F2* c.344–979T>A, intron variant) associated with bleeding risk, particularly with ticagrelor
Bourgeois et al. [34]	Genome wide association study for 1478 patients	478 cases (endoscopically confirmed PUD with NSAID use within 3 months) and 495 controls (endoscopically confirmed PUD defined as mucosal breaks ≥ 3 mm in diameter at unspecified location without history of NSAID use within 3 months)	Various NSAIDs and aspirin	Meta-analysis data proved genome-wide significant association for *EYA1* rs12678747
Xu et al. [35]	Subgroup analysis including 372 patients from the original PRINCE trial (675 patients)	Hospitalized patients getting patients getting ticagrelor + aspirin or clopidogrel + aspirin within 24 h of TIA or stroke	Aspirin	Genetic polymorphisms in *PEAR- 1* affect platelet function and bleeding
Cho et al. [36]	Korean prospective case–control study examining association between *IL- 1β* genotype and peptic ulcer disease (PUD) in aspirin users	23 cases with aspirin use and endoscopically confirmed PUD and 25 controls with aspirin use but no ulcers	Aspirin use (case vs control)	*IL-1β − 581* and *IL-1β − 1061* genotypes associated with increased risk of peptic ulcer in aspirin users
Forgerini et al. [37]	Case control study of genetic risk factors for upper GI bleeding in patients with NSAID use	200 cases with UGI bleeding and 700 controls	NSAIDs (piroxicam, celecoxib, naproxen, aceclofenac, indomethacin, diclofenac, ibuprofen) and aspirin (300 mg)	CYP2C9*3 and *VKORC1* genotypes associated with increased risk of UGI bleeding among NSAID users, particularly in higher NSAID doses

Most studies focused on *CYP2C9* variants *2 and *3 that alter the pharmacokinetics of NSAIDs and increase the risk of upper GI bleeding/ulceration [27–29]. Pilotto et al*.* [27] studied 26 patients with NSAID-related upper GI bleeding and found that the CYP2C9*1/*3 and CYP2C9*1/*2 genotypes were linked to a higher bleeding risk, but CYP2C9*2/*3 was not. After adjusting for Cumulative Illness Rating Scale-Comorbidity Index, only the CYP2C9*3 allele showed a significantly higher risk (OR, 7.3; 95% CI, 2.058–26.004), while no association was found for CYP2C9*2 (OR, 2.0; 95% CI, 0.634–6.317). Martinez et al*. *[28] compared NSAID-related upper GI bleeding cases with controls who used NSAIDs without adverse effects. They found that carriers of variant alleles of *CYP2C9* had a higher risk of bleeding. Specifically, the OR for carriers of variant alleles was 2.60 (95% CI 1.10–6.19). CYP2C9*2 was significantly associated with bleeding (OR 1.92, *p* = 0.009), while CYP2C9*3 was not (OR 1.01, *p* = 0.987). The study also noted that infection with *Helicobacter pylori* could confound the results for CYP2C9*3. Figueiras et al*. *[29] conducted a multi-center study with 577 GI bleeding cases and 1,343 controls. The study found that patients taking NSAIDs above 0.5 WHO daily defined dose [38] and carrying the CYP2C9*3 allele had a significantly higher risk of GI bleeding (adjusted OR 18.07, CI 6.34–51.53). No significant association was found with the CYP2C9*2 allele.

Variants in genes regulating prostaglandin synthesis or function, as well as mucosal healing, have also been implicated in NSAID-related GI toxicity (upper GI bleeding and ulceration). Kraus et al*.* [30] found that certain genetic variants were linked to higher GI toxicity in Israeli patients on celecoxib, using a subset of the PreSAP trial with 117 participants. The *Epidermal growth factor-like receptor (EGFR)* rs2072454 SNP was associated with increased GI toxicity (OR = 2.43, *p* = 0.04), while celecoxib users with *Arachidonate 15-lipoxygenase (ALOX15)* rs2255888 (OR = 3.49, *p* = 0.02) and *Prostaglandin E receptor 4 (PTGEP4)* variants (rs4133101, OR = 5.52, *p* = 0.04; rs13186505, OR = 3.35, *p* = 0.03) had even higher risks. These findings suggest that disruptions in COX-2 and related pathways contribute to GI toxicity.

Multiple other genes and genetic variants have been reported to be associated with gastrointestinal toxicity and NSAID use. Shiotani et al*. *[31] found the *Angiotensinogen (AGT)* c.−20C (rs505) genotype was more prevalent in aspirin users with bleeding ulcers. *The Angiotensin II receptor type 1* (*AT1R)* c.−521CC genotype was linked to peptic ulcers in non-ACE inhibitor/ARB users. They also identified variants in other genes (*CYP4F11*, *CYP2D6*, *CYP24A1*, *GSTP1B*) to be associated with small bowel bleeding [32]. Tatarunas et al*. *[33] found *CYP4F2*T allele (rs3093135, c.344–979T>A) increased bleeding risk in aspirin and clopidogrel or ticagrelor users. Bourgeois et al*. *[34] linked *EYA transcriptional coactivator and phosphatase 1* (*EYA1)* SNP (rs12678747, intronic variant) to aspirin-related peptic ulcers, and Xu et al*. *[35] found the *Platelet endothelial aggregation receptor 1* (*PEAR1)* A allele rs12041331 (c.− 9- 3996G>A, intronic variant) is linked to aspirin bleeding risk. Cho et al*. *[36] associated *IL-1β* gene variants with aspirin-related peptic ulcers, and Forgerini et al*. *[37] linked not only CYP2C9*3 but also *Vitamin K epoxide reductase complex subunit 1 (VKORC1)* variants to NSAID-related upper GI bleeding.

The reviewed literature noted above also highlights the need for more genetic studies particularly focused on NSAID-induced small bowel ulcerating disease. Another key resource regarding NSAID injury is the Clinical Pharmacogenetics Implementation Consortium (CPIC) Guidelines, which offer therapeutic recommendations for NSAID use based on the *CYP2C9* genotype. While a detailed discussion of these guidelines is beyond the scope of this review, we recommend consulting the manuscript by Theken et al., which provides a comprehensive overview of the CPIC guidelines [39]. These guidelines are periodically updated and can be accessed at www.cpicpgx.org/guidelines/.

## Cryptogenic Multifocal Ulcerous Stenosing Enteritis (CMUSE) & Chronic Non-Specific Ulcers of the small intestine (CNSU) [40, 41]

The exact prevalence of CMUSE and CNSU is unknown, but these diseases are extremely rare, with the latter occurring mainly in the Japanese population. However, the lack of small bowel endoscopies in some regions of the world or awareness about these diseases could contribute to their low prevalence. The signs and symptoms usually start in childhood or early adulthood. Recurrent ulcers cause small bowel bleeding and anemia, whereas strictures give rise to obstructive symptoms and even inadvertent capsule retention. Patients with CNSU develop skin and bone changes such as pachydermia, primary hypertrophic osteoarthropathy, and nail changes, unlike CMUSE. On endoscopy and radiology, the ulcers and strictures appear similar to NSAID enteropathy and CD. Histology of CMUSE and CNSU is also nonspecific (Fig. 1).

Current diagnostic criteria for CMUSE or CNSU are:

1. Unexplained small-bowel strictures
2. Superficial ulcers restricted to the mucosa and submucosa
3. Chronic or relapsing ulcerative stenosis and abdominal pain
4. No signs of systemic inflammation
5. Persistent and occult blood loss from the GI tract, except during bowel rest or postoperative period.

### Genetics of CMUSE/CNSU [40, 41]

Over the past two decades, genetic studies have linked CMUSE and CNSU to two important Prostaglandin (PG) mediated enteropathies. The pathophysiology of CMUSE needs to be better understood. It was previously considered to be some form of vasculitis. Based on the genetic findings, it is now considered to be due to variants in *PLA2G4A* gene, which is responsible for encoding phospholipase A2 (PLA2), which cleaves membrane phospholipids to arachidonic acid. Hence, these patients have deficient PG and impaired intestinal healing.

Similarly, CNSU, also known as chronic enteropathy associated with *SLCO2A1* (CEAS), has loss of function mutation in *SLCO2A1*, a solute anion transporter family protein responsible for PG reuptake into various body cells. It is unknown why these genetic mutations in PG synthesis and handling affect small bowel exclusively. Also, not all reported cases of CMUSE harbor *PLA2G4A* mutation. Lack of PGs can impair mucosal healing but the primary trigger for initial injury remains unknown.

## Conclusion

In conclusion, while the growing body of literature has provided valuable insights into the genetic associations with small bowel ulcerating diseases, studies specifically aimed at exploring the role of genetics in distinguishing between these conditions are still limited. There is a critical need for research focused on leveraging genetic variations to enhance diagnostic accuracy and differentiate between diseases such as CD, NSAID enteropathy, and CMUSE/CNSU. Addressing this gap in research would significantly improve clinical outcomes and reduce the risk of misdiagnosis, ultimately leading to more effective management of these complex conditions.

## Key References

- Huang H, Fang M, Jostins L, Umićević Mirkov M, Boucher G, Anderson CA, et al. Fine-mapping inflammatory bowel disease loci to single-variant resolution. Nature. 2017;547(7662):173–8. 10.1038/nature22969.
  - ○ The first study to successfully refine the genetic loci associated with IBD to a single-variant resolution. This fine-mapping increased the resolution of prior association studies, which often only identified broad genomic regions but lacked the specificity to pinpoint the exact genetic drivers.
- Sazonovs A, Stevens CR, Venkataraman GR, Yuan K, Avila B, Abreu MT, et al. Large-scale sequencing identifies multiple genes and rare variants associated with Crohn's disease susceptibility. Nat Genet. 2022;54(9):1275–83. 10.1038/s41588-022-01156-2.
  - ○ The authors studied more than 30,000 CD exome sequenced patients and compared them with 80,000 population controls. Besides the large cohort, the authors focussed on identifying rare coding variants that would provide more informative cues to disease pathogenesis than GWAS identified non-coding variants.
- Shiotani A, Murao T, Fujita Y, Fujimura Y, Sakakibara T, Nishio K, et al. Novel single nucleotide polymorphism markers for low dose aspirin-associated small bowel bleeding. PLoS One. 2013;8(12):e84244. 10.1371/journal.pone.0084244.
  - ○ The only study to identify a SNP associated with low dose aspirin (NSAID) and small bowel bleeding.
- Moreels TG, Singh A. Updates on the diagnosis and management of cryptogenic multifocal ulcerative stenosing enteropathy (CMUSE) and non-steroidal enteropathy. Best Practice & Research Clinical Gastroenterology. 2023;64–65:101,847. 10.1016/j.bpg.2023.101847.
  - ○ Study provides best comprehensive review and current knowledge about the rare disorders CMUSE and CNSU.

## Data Availability

No datasets were generated or analysed during the current study.
